# In-Person Versus eHealth Mindfulness-Based Intervention for Adolescents With Chronic Illness: Protocol for a Randomized Controlled Trial

**DOI:** 10.2196/resprot.7700

**Published:** 2017-11-27

**Authors:** Nicholas Chadi, Miriam Kaufman, Elli Weisbaum, Catherine Malboeuf-Hurtubise, Sara Ahola Kohut, Christine Viner, Jake Locke, Dzung X Vo

**Affiliations:** ^1^ Division of Adolescent Medicine, Department of Pediatrics Hospital for Sick Children University of Toronto Toronto, ON Canada; ^2^ Department of Pediatrics Boston Children's Hospital Harvard Medical School Boston, MA United States; ^3^ Institute of Medical Sciences University of Toronto Toronto, ON Canada; ^4^ Research Group on Mindfulness Montreal, QC Canada; ^5^ Department of Educational Sciences Université du Québec en Outaouais Gatineau, QC Canada; ^6^ Medical Psychiatry Alliance Hospital for Sick Children Toronto, ON Canada; ^7^ Department of Psychiatry University of Toronto Toronto, ON Canada; ^8^ Department of Pediatrics Downstate Medical Center State University of New York New York, NY United States; ^9^ Department of Child and Adolescent Psychiatry British Columbia Children's Hospital University of British Columbia Vancouver, BC Canada; ^10^ Division of Adolescent Health and Medicine, Department of Pediatrics British Columbia Children's Hospital University of British Columbia Vancouver, BC Canada

**Keywords:** mindfulness, meditation, chronic illness, adolescent, eHealth, randomized, protocol

## Abstract

**Background:**

Eight-week mindfulness-based interventions (MBIs) have a beneficial impact on mental health and well-being in adolescents with chronic health conditions. Usually delivered in person in a group setting, these programs are difficult to access for teens with disabilities or who do not have in-person MBIs available in their communities.

**Objective:**

This paper outlines the rationale, development, and design of a randomized controlled trial comparing the effects of an MBI delivered in person or via eHealth in adolescents with a chronic illness. Quantitative outcomes will include mindfulness skills acquisition (primary outcome), effects of the MBI on self-reported mood, anxiety, self-esteem, illness perception, and physiological stress (via salivary cortisol), and qualitative outcomes will include individual practice, participant appreciation, and adaptation of the MBI for eHealth.

**Methods:**

This is a randomized noninferiority mixed methods study comparing 2 MBI arms: in-person and eHealth. Participants are eligible to participate if they are aged 13 to 18 years, have a diagnosis of chronic medical condition, live close enough to the recruitment hospital to participate in the in-person arm of the study, and are currently followed by a health care provider. Each participant will receive an adapted 8-week MBI delivered either in person at a tertiary pediatric hospital or via a secure audio-visual platform allowing group interactions in real time. Groups will be facilitated by 2 experienced mindfulness providers. Quantitative and qualitative data will be collected through standardized research questionnaires administered via a secure, youth-friendly online platform and through semistructured interviews, participant log books, facilitator log books, and salivary cortisol analysis. Qualitative data will be analyzed using a grounded theory model.

**Results:**

Data collection is currently underway. Data analysis, manuscript writing, and additional publications are expected to be completed in the winter and spring of 2018.

**Conclusions:**

Based on previous results from in-person trials conducted in adolescents and eHealth trials conducted in adults, we anticipate that both modes of delivery will significantly improve mindfulness skills acquisition, mood, anxiety, self-esteem, illness perception, and stress and that the magnitude of the effects will be correlated to the level of home practice. We predict that participants in both arms will show similar levels of home practice and that both modes of delivery will have high levels of feasibility and acceptability. If successful, this study could provide evidence for the use of eHealth in the delivery of 8-week MBIs in clinical adolescent populations, potentially increasing availability to MBIs for a large group of youth with mobility issues or living away from large urban centers.

**Trial Registration:**

ClinicalTrials.org NCT03067207; https://clinicaltrials.gov/ct2/show/NCT03067207 (archived by WebCite at http://www.webcitation.org/6v4ZK8RBH)

## Introduction

Chronic illnesses in adolescents are common and have significant repercussions on normal development and well-being [1]. For instance, adolescents with chronic health conditions may experience challenges in negotiating the tasks of adolescents, such as the achievement of independence, and often report frustration with the management of their condition [2]. It is estimated that approximately 30% of adolescents have at least one moderate or severe chronic health condition such as asthma, depression, or diabetes [3]. Chronic illness can impact mental health, brain development, sleeping patterns, social functioning, school performance, and family relationships [4]. Emerging research shows that teen-adapted mindfulness-based interventions (MBIs) can provide important benefits in these areas and be a useful adjunct in the treatment of chronic health conditions [5]. Drawing from meditation practices rooted in a number of Buddhist traditions, most contemporary forms of mindfulness meditation studied in the setting of clinical research are presented in a secular context and seek to promote purposeful and nonjudgmental awareness of one’s thoughts, feelings, and bodily sensations [6]. Research performed in the elementary and high school systems has shown that MBIs have a positive impact on mood, anxiety, and resilience, as well as cognitive and social-emotional development [7-9]. High-quality data in clinical youth populations is emerging and suggests similar results [10-17].

There has been a recent increase in the number of moderated and self-directed eHealth resources promoting well-being in adults and youth [18]. Research has shown that MBIs for adults with chronic diseases, delivered via phone or the Internet, have excellent feasibility and positive effects on mood and life satisfaction [19-21]. Building on the results of promising in-person pilot trials showing high validity and acceptability among adolescent study participants [11,12,15,22-28], our research will address the critical barrier of access to MBIs by exploring a new avenue for youth—eHealth—with the objective of offering equal opportunities to adolescents with reduced mobility due to illness or disability or who live in areas making it difficult or impossible for them to access in-person mindfulness groups.

To our knowledge, to date, the feasibility, acceptability, and effectiveness of youth-adapted in-person and eHealth mindfulness interventions has not been compared head-to-head in a clinical youth population. There is also a clear need for the development and adoption of eHealth interventions to support children and youth in challenging contexts such as chronic illnesses. Adolescents living in rural or underserved areas have a lower degree of access to quality care, which can adversely impact their well-being [29]. For example, a recent systematic review of psychological interventions for adolescents and young adults living with chronic illnesses emphasizes the importance of conducting high-quality randomized studies exploring new ways to support young people with chronic health conditions [30].

This article outlines the rationale, development, and design of a randomized controlled trial comparing the effects of an MBI delivered in person or via eHealth in adolescents with a chronic illness. Our primary aim is to compare the impact of an MBI for adolescents with chronic health conditions delivered either in person or via eHealth on mindfulness skills acquisition with the overarching goal of setting the stage for a larger multicenter trial within this population. The secondary objectives are to gather quantitative data on the effects of the MBI related to mood, anxiety, self-esteem, illness perception, stress (via salivary cortisol), and qualitative data on individual practice, adaptation of the MBI for eHealth, and participant experience. We hypothesize that the eHealth mode of delivery will be noninferior to the in-person mode of delivery and that the feasibility and acceptability of the MBI will be high in both groups.

## Methods

### Overview

This trial will use a mixed methods approach gathering (1) quantitative self-report data via validated questionnaires, (2) qualitative data on the participant’s and facilitator’s individual experience of the program using logbooks and semistructured interviews, and (3) biological data in the form of salivary cortisol measures as a surrogate marker for stress. Our mixed methods approach has been designed to help us better understand the potential impact, benefit, and effectiveness of an MBI on adolescents with chronic illness from both a clinical and experiential perspective.

### Population

Adolescents aged 13 to 18 years with a diagnosis of chronic illness or disability receiving care at a large pediatric tertiary care hospital in Toronto, Canada, are eligible to participate. Teens with a condition lasting more than 1 year with associated functional impairment or requiring ongoing medical care will be considered to have a chronic illness [31]. Multiple clinics within the hospital have been targeted for recruitment including but not limited to Neurology, Neurosurgery, Rheumatology, Gastroenterology, Endocrinology, Hematology, and Medical Psychiatry. Examples of chronic illnesses that eligible patients may have include thalassemia, sickle cell anemia, inflammatory bowel disease, migraines, epilepsy, juvenile rheumatoid arthritis, lupus, and somatic symptom disorder.

Patients diagnosed primarily with a mental health condition such as depression and anxiety are eligible to participate provided they also have a chronic physical symptom (eg, chronic pain, headaches, abdominal pain). Participants must be fluent in English and have the intellectual capacity to provide independent consent and complete research questionnaires. To participate in the study, potential participants will need to live close enough to the recruitment hospital to commit to 8 in-person sessions delivered at the referring hospital. Youth who have previously participated in an MBI will be eligible to participate. Exclusion criteria include acute suicidal ideation unknown to or unaddressed by the referring health care provider and developmental disability such as moderate-to-severe intellectual disability impacting ability to take part in the study.

### Recruitment and Enrollment

Recruitment for this study will primarily take place in outpatient clinics of a tertiary care pediatric hospital when patients arrive for their scheduled initial or follow-up appointments. Participants will be referred for inclusion in the study by a member of their health care team after discussion with the patient and family. Nurse practitioners and social workers will also screen clinic lists to proactively identify potentially eligible participants. Brief presentations of the study will occur at divisional rounds and with small groups of health providers to increase awareness about the study. Posters and brochures will be distributed throughout clinics and offices.

Recruitment will occur until a convenient sample size of 60 participants is reached or until a maximum of 3 months of active recruitment has occurred (whichever happens first). Based on the results of a pilot study performed by our research team with 19 female adolescents with chronic pain [15], we anticipate that a period of 3 months will be sufficient to enroll the targeted number of participants (average enrollment rate: 5 participants per week).

A research assistant (CV) will contact every interested participant by phone to confirm eligibility and provide additional information about the sessions. If the participant is deemed preliminarily eligible and is interested in the trial, an intake meeting will be scheduled. The meeting will take place in person or via a secured password-protected online platform (for participants who are unable to meet in person). The platform, called Zoom Video Conferencing (Zoom Video Communications Inc), will allow individual or group audiovisual meetings through a mobile phone, tablet, or computer. Meetings (both in person and via the online platform) will be led by a member of the research team with expertise in mindfulness-based cognitive therapy (MBCT) and will include an introductory video on mindfulness and a detailed explanation of the MBI, study design, and differences between the in-person and eHealth arms. A discussion about the importance of committing to individual practice and maximal attendance for mindfulness sessions will take place. The limits of confidentiality will be discussed with emphasis on sessions being a safe space where participants feel they can express themselves freely and, if placed in an eHealth group, where they will be in view of their webcam at all times, without other people in the room. Consent will be discussed with the research assistant (CV) at the end of the information meeting. Participants will be notified that both in-person and eHealth sessions will be video-recorded for internal validity purposes, and videos will only be accessed by a team member with extensive mindfulness meditation experience (coauthor CMH).

### Attrition and Compliance

Based on the results of a pilot study by Chadi and colleagues [15], we expect attrition rates to be low (between 10% and 20%) and compliance (attendance for mindfulness sessions) to be high, with a mean attendance rate of 6 to 7 sessions out of 8. To our knowledge, no study examining 8-week MBIs delivered via eHealth in adolescents is currently available. Extrapolating from studies conducted in adults [22,28], we anticipate that attrition rates will be comparable for in-person and eHealth groups.

Participants having completed at least 4 of the 8 mindfulness sessions will be considered to have attended sufficiently to be included in data analysis. Pilot data on individual home practice suggests that participants will be practicing between 2 and 10 times per week for a median duration of 8 minutes per practice [15].

### Study Design

**Figure 1:**
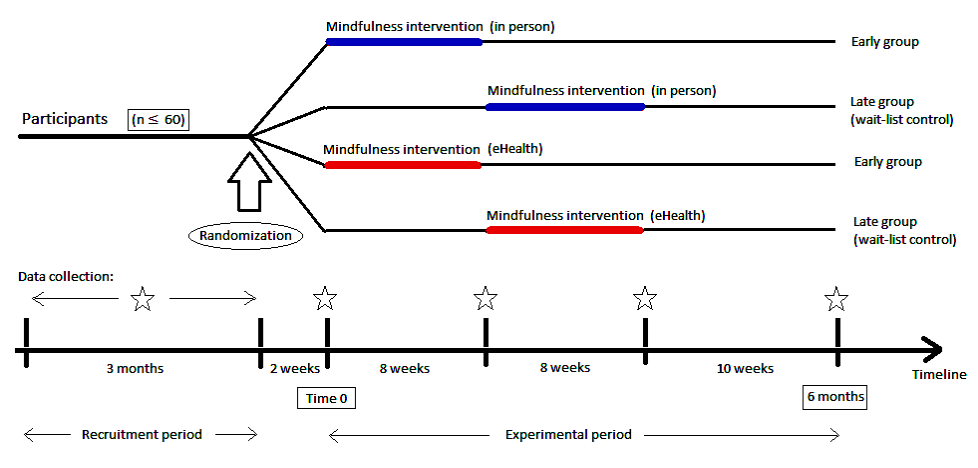
Experimental design of a randomized longitudinal trial comparing in-person versus eHealth delivery of an 8-week mindfulness-based intervention for adolescents with chronic illness.

The study will be conducted as a randomized noninferiority trial comparing 2 arms: an in-person and an eHealth arm, as shown in Figure 1. Each arm will consist of 2 groups with a target of 15 participants each: 1 early intervention group, 1 wait-list group. An additional feasibility arm will take place if the targeted number of study participants (60) is not reached and will be offered to participants who are interested in participating in the study but are not able to commit to the in-person mode of delivery. It will consist of 2 groups, 1 early eHealth group and 1 wait-list eHealth group.

All participants will receive the mindfulness intervention either in person or via eHealth by the end of the 6-month study period. No participants will be excluded from participation based on financial or technical limitations. Should participants not have access to an Internet connection and a mobile phone, tablet, or computer at home, the research assistant will speak with participants and their parent or guardian to explore alternate available resources (ie, connecting at school, at a local library, through a local health center, at a relative’s or friend’s home).

**Table 1:** Overview of data collection.

Time point/group	Baseline	Time 0	Week 8	Week 16	6 months
Early groups: experimental arm (distance or eHealth). MBI^a^ begins at time 0.	Background data; Research questionnaires	Salivary cortisol; Participant and facilitator logs (weekly for 8 weeks)	Salivary cortisol; Research questionnaires; Semistructured interviews	Research questionnaires	Research questionnaires
Wait-list control groups: experimental arm (distance or eHealth). MBI begins at week 8.	Background data; Research questionnaires	—	Salivary cortisol; Research questionnaires; Participant and facilitator logs (weekly for 8 weeks)	Salivary cortisol; Research questionnaires; Semistructured interviews	Research questionnaires

^a^MBI: mindfulness-based intervention.

At the end of the recruitment period, once eligibility criteria have been verified, randomization will take place to separate participants in the 4 different groups within the in-person and eHealth arms of the study.

### Randomization and Blinding

This study will be a single-blinded study (data analysis only). Participants will be coded using Arabic numerals. A research assistant (CV) will receive all questionnaires and saliva samples. Mindfulness facilitators will be unaware of participant identification numbers. Participants will be assigned to 1 of 4 experimental groups following a computer-generated randomization list (permuted block design, using blocks of 2 or 4). No stratification will be used. The allocation sequence will be generated by a statistician not otherwise involved in the project and passed on to a research assistant after patient identification data has been collected (ensuring that those participating in data analysis are kept blind to the allocation of each participant). Allocation sequence will be password-protected and only accessible to the statistician and research assistant. The research assistant will contact participants by phone to inform them to which group they have been randomized.

### Data Collection

Data collection will take place at baseline (the day of the intake meeting), immediately before and after the MBI, and at the end of the 6-month study period, as detailed in Table 1.

All participants will provide background information at intake including age, birthdate (month/year), grade at school, past medical history, medication, and previous exposure to contemplative sciences (including yoga, meditation, and mindfulness). Participants will provide data through research questionnaires and salivary cortisol as well as individual semistructured interviews with the research assistant as detailed in Table 2. Participants will also provide information about individual practice using electronic participant logs during the 8 weeks of the MBI and when completing research questionnaires at the end of the sixth month of the study period. Data from research questionnaires and participant logs will be collected via the TickIT platform (GASQ Service GmbH), a youth-friendly, secure, digital assessment platform, via computer, tablet, or mobile phone [32]. Data compilation will take place through the TickIT platform as well.

**Table 2:** Summary of primary and secondary outcome measures.

Outcome	Measurement	Description	Length
Mindfulness skills acquisition (primary outcome)^a^	Mindful Attention Awareness Scale for Adolescents (MAAS-A) [41]	14-item scale evaluating level of mindfulness as a quality of attention informed by an awareness of the present experience	Approximately 3 min
Illness perception^a^	Illness Perception Questionnaire (brief) [47]	9-item measure of health-related quality of life for adolescents including those with acute and chronic health conditions. Provides an overview of how the illness is affecting overall level of functioning	Approximately 3 min
Mood and anxiety^a^	Depression Anxiety Stress Scale (DASS-21) [48]	21-item scale assessing mood and anxiety symptoms in adolescents based on self-evaluation of symptoms in the past 7 days	Approximately 5 min
Self-esteem^a^	Rosenberg Self Esteem Scale [49]	10-item scale assessing self-esteem and overall satisfaction with life	Approximately 3 min
Stress (biological marker)	Salivary cortisol levels [42]	Self-collected saliva samples using cotton swabs (Salivettes). Analysis by immunoassay	1 minute per sample
Individual practice	Data reported in participant log books	Participants will be encouraged to detail individual home practice in a digital log book noting frequency, duration, and type of practice	Approximately 5 min per week
Participant appreciation	Verbatim transcription of semistructured interviews	Individual interviews with study participants will be conducted at the end of the 8-week MBI^b^ using the Zoom eHealth platform	15 to 30 min
Facilitator feedback	Data reported in facilitator log books	Facilitators will use pre- and postsession log books to detail impressions and adaptations of the MARS-A^c^ program for the eHeath platform	Approximately 15 min per entry

^a^All measurement scales included in this table have been validated in clinical youth populations and have shown favorable psychometric profiles (Cronbach alpha>.80).

^b^MBI: mindfulness-based intervention.

^c^MARS-A: Mindful Awareness and Resilience Skills for Adolescents.

All interviews will take place through the Zoom electronic audio-visual platform and will be led by a female research assistant, coauthor CV (MD). The research assistant will use an interview guide adapted from a study led by coauthor SAK [33]. She will be encouraged to take field notes during the interviews and will have received individual training in conducting semistructured interviews by coauthors EW (MEd) and SAK (PhD), who both have experience leading semistructured interviews and focus groups with youth. Participants will complete the interview in a private room, at home, with no other nonparticipants or researchers present and will be informed that the research assistant is a medical doctor, has limited experience with mindfulness, and has interest in understanding the effects of mindfulness in adolescents with chronic medical conditions. The research assistant will have met with each participant at baseline either in-person or virtually through the Zoom platform for a recruitment meeting. Recordings will be password-protected and only the research assistant (who will also be transcribing the recordings) will have access to them. Transcriptions will be de-identified using a patient code available only to the research assistant. Transcripts will be returned to participants for comment and correction if there is ambiguity at the time of analysis. Data will be analyzed using a grounded theory model [34] until data saturation is reached. Data for secondary outcomes will be collected for all participants. It is expected that research questionnaires will take 15 to 20 minutes to complete.

Saliva samples for cortisol analysis will be drawn at wake-up, at 12:00 PM, at 5:30 PM, and at 7:00 PM on the first and eighth (last) MBI session to capture fluctuations in cortisol levels throughout the course of the day. Participants will receive a reminder phone call the day before each saliva sampling occurs. This will allow for the comparison of pre-post cortisol level differences between participants in the in-person and eHealth arms. Participants in the eHealth arm will be provided with prepaid postage for return of saliva samples, which they will be able to send from home. It has been shown that saliva samples can be exposed to room or exterior temperatures for at least 72 hours without affecting cortisol values [35]. Once received, saliva samples will be kept frozen until they are sent to a specialized laboratory for analysis by enzyme immunoassay [36].

### Intervention

The intervention will consist of the 8-week Mindful Awareness and Resilience Skills for Adolescents (MARS-A) program [23,37], an evidenced-informed in-person MBI adapted specifically for youth with chronic health conditions. This program was developed by coauthors DV and JL at British Columbia Children’s Hospital and the University of British Columbia. MARS-A is based on 3 established evidence-based MBIs for adults and adolescents: mindfulness-based stress reduction (MBSR) [6,38], mindfulness-based stress reduction for teens (MBSR-T) [13], and MBCT [39,40]. The content of the program will be the same for in-person and eHealth groups with only minor adjustments for the eHealth group to allow all participants to remain in view of their Web camera during the sessions. All participants will be offered eight 90-minute weekly evening sessions—this represents an adaptation from adult MBSR and MBCT sessions which are typically 150 minutes in length—led by 2 experienced mindfulness providers with committed daily meditation practices (for this study, coauthor NC with the assistance of coauthor EW), who have been trained in facilitating MARS-A and who will also adapt the program for the online group. Also, while the MARS-A program usually includes a silent half-day retreat, this will not be included in the intervention so as not to disadvantage participants in the eHealth group for whom it might not be feasible to create a silent environment for a longer period of time. Sessions will include short meditation practices, breathing exercises, guided discussion (mindful inquiry), and mindful movements adapted to the context of chronic illness. All participants will also receive a copy of a self-help book for adolescents, *The Mindful Teen: Powerful Skills to Help You Handle Stress One Moment at a Time* [37] (by coauthor DV), to encourage regular practice and maintenance of newly acquired mindfulness skills. Every session will start with a brief review of home practice and will close with a discussion of the home practice for the following week. Participants will receive a weekly reminder email before each session to encourage them to complete home practice exercises and to remind them about the date and time of the next in-person or online mindfulness session. A summarized thematic overview of the MARS-A program can be found in Table 3.

Participants in the in-person groups will meet at the hospital in a designated teen-friendly room containing chairs and yoga mats. Sessions will take place once a week after school from 5:30 PM to 7:00 PM. Participants will have a 5-minute break at the middle of each session, and a snack will be provided.

Participants in the eHealth groups will be encouraged to find a quiet room with as little outside noise and distraction as possible. They will be required to have access to the Internet, a desktop or laptop computer equipped with a webcam or a tablet or mobile phone with webcam function. Participants and facilitators will be connected using the secure password-protected audio-visual platform Zoom Video Conferencing allowing up to 50 different computer connections. With this interface, participants and instructors can see and hear each other and interact in real time. This interface also allows for audio and video recording of mindfulness sessions and allows hosts to restrict access to meeting members who might not behave appropriately during sessions. All in-person and eHealth mindfulness sessions will be recorded and reviewed by a clinical psychologist with experience and expertise in mindfulness for adolescents (coauthor CMH) to ensure delivery of similar content between groups.

**Table 3:** Thematic overview of the Mindful Awareness and Resilience Skills for Adolescents curriculum.

Week	Theme	Content
1	Stress, Depression and Health—Introduction to Mindfulness for Adolescents	Introduction to mindfulness and group expectations Introduction to stress and depression Group and home practices: breathing practice, sitting meditation, and the body scan
2	Foundations of Mindfulness	Developing a practice Key elements of mindfulness Group and home practices: body scan, mindful eating, sitting meditation
3	Informal Mindfulness Practice and Gratitude	Introduction to informal mindfulness practice Gratitude and pleasant events Group and home practices: mindful movement, 3-minute breathing space, walking meditation
4	Unpleasant Experiences, Physical Sensations, Physical Pain	Discussion: making peace with pain, suffering, and unpleasant physical sensations Group and home practices: mindful stretching, 3-minute breathing space, body scan
5	Seeing Thoughts as Thoughts	Cognitive exercise: thought distortions Discussion: mindfulness of thoughts, rumination Group and home practices: sitting meditation, short breathing practices
6	Handling Emotions	Discussion: handling emotions Group and home practices: mindful stretching, walking meditation, 3-minute coping space
7	How to Best Take Care of Yourself	Discussion: taking care of yourself Developing an action plan for future practice Group and home practices: loving-kindness meditation, sitting meditation, 3-minute coping space
8	Continuation of Practice	Course review and evaluation Discussion: maintaining a practice End of course celebration Group practices: mindful stretching, silent sitting, 3-minute breathing space

All participants will be invited to complete daily mindfulness meditation exercises at home. Participants will be asked to fill a practice log tracking the type, duration, and frequency of individual mindfulness home practice at the beginning of each mindfulness session using the TickIT online platform used for research questionnaires.

### Outcome Measures

The primary outcome measure for this study is mindfulness skills acquisition, assessed by the Mindful Attention Awareness Scale for Adolescents (MAAS-A) [41]. This 14-item Likert-type scale has been previously validated in large samples of youth with chronic health conditions. Secondary outcomes will include quantitative and qualitative measures as detailed in Table 2: illness perception, mood and anxiety, self-esteem, cortisol levels (biological marker for stress) [42], individual practice, personal experience during the program, and facilitator feedback about adaptation of the MARS-A curriculum for the eHealth platform. The authors chose salivary cortisol levels as the biological marker for this study because of the possibility of using a noninvasive sampling method (ie, in comparison with blood tests) and based on promising, yet inconsistent reports of the effects of MBIs on cortisol levels and recommendations for future study [43]. They also chose cortisol in an attempt to reproduce the findings of a pilot study by Chadi and colleagues [15] where reductions in cortisol levels during a mindfulness session had been shown to increase significantly pre- and post-MBI in a similar population of youth with chronic pain.

### Power Calculations

Sample size was determined on a realistic estimate based on recruitment rates from previous pilot studies. We estimate that 3 months will suffice to recruit 60 participants. With half the participants in the in-person groups and half the participants in the eHealth groups, the study will have 80% power of confirming noninferiority of the eHealth mode of delivery with a margin of 0.45 points on the MAAS-A scale. This margin reflects the change in MAAS-A scores found in the initial validation study of the MAAS in adolescents using an in-person program [41]. Unfortunately, the convenient sample size of 60 participants used for this study will not allow sufficient power to demonstrate noninferiority of secondary outcomes assessed by standardized questionnaires, based on recent mindfulness studies using these measures in adolescent populations [44,45].

### Data Analysis

Quantitative and qualitative data analysis will take place as described in Table 2. All quantitative analyses will be conducted using SPSS 24th edition (IBM Corp). Descriptive statistics will be used to identify participant demographics and disease characteristics. Quantitative self-report data will be analyzed using 1-way analyses of variance and analyses of covariance as well as paired *t* tests to assess pre-post MBI changes and to compare outcomes in the in-person versus eHealth arms as well as between the early versus wait-list control groups. A margin of 0.45 will be used as a prespecified noninferiority margin. For the primary outcome (mindfulness skills acquisition), the noninferiority question will be based on a confidence interval of 95% (2-sided) in relation to the noninferiority margin for the different treatment groups.

Transcribed interviews will be analyzed using inductive content analysis [46]. Inductive qualitative content analysis allows for a systematic classification of the data to identify categories based on patterns. By not imposing a preexisting coding schema, inductive content analysis allows for novel insights and understanding from the perspectives of participants grounded in their experiences of attending the MBI either in-person or via eHealth. Coding will be completed in a phased approach using NVivo software (QSR International Pty Lmd). In phase 1, 3 investigators (NC, EW, CV) will review 2 transcripts independently using an inductive open coding approach and then meet to develop codes based on group consensus. Codes will be discussed such that the 3 investigators agree upon (1) the major and minor themes that codes represented and (2) codes could be assigned to meaning units (phrases to several sentences). In phase 2, the same 3 investigators will code an additional 2 transcripts independently using the developed codes and meet again to discuss and confirm the coding structure. In phase 3, 2 investigators (NC, EW) will independently code the remaining transcripts. Investigators will be in contact to discuss findings should any new codes emerge from the data. Should any new codes arise, all previously coded transcripts will be reviewed for new codes. If there are disagreements among the 2 investigators, the third investigator will code to arrive at a consensus and ensure consistency with original coding structure. In phase 4, the 3 investigators will meet to collapse all resulting codes into categories. Given the ongoing communication and coding to consensus, interrater reliability will not be calculated. Major and minor themes will be presented using participant quotes (identified by participant number) in the final account of study results.

### Ethics and Participant Safety

This study has received full approval from the Research Ethics Board of the Hospital for Sick Children in Toronto and has undergone a local institutional scientific review. To our knowledge, the MARS-A program does not pose any significant risks to the physical and psychological safety of participants, although some participants might experience temporary unpleasant physical, emotional, or psychological experiences while discussing symptoms related to pain, disability, or a chronic health condition. All participants will be expected to continue medical treatment as usual with their health care provider for the duration of the intervention. Activities requiring movement, such as mindful movement and walking, will be tailored to the capacities and needs of each participant and slightly adapted for the eHealth group to allow participants to stay in view of their Web camera. There is no anticipated risk with the testing of salivary cortisol. Questionnaires that will be used have been validated in studies involving large numbers of adolescents. It is possible that study participants may feel some mild degree of stress and anxiety when asked about depressive or anxious symptoms while completing the questionnaires. The process of expert mindful inquiry and facilitation itself is designed to help participants handle distress in the supportive environment of the mindfulness intervention [50]. At any time during the study, if participants disclose symptoms suggesting a new physical or psychological condition, they will be connected with their referring provider or with an emergency contact identified by the participant during the preparticipation meeting. Confidentiality will be addressed to fulfill the requirements of the local institutional review board.

All parking costs will be reimbursed at the end of the study period, whether participants complete the study or not. Participants who complete the project and submit all research questionnaires will receive a Can $20 (US $16) gift card at the end of the study period. Participants will also receive a certificate of completion of the MARS-A curriculum at the end of the study period regardless of the number of mindfulness sessions attended.

## Results

Data collection is currently underway. Data analysis, manuscript writing, and additional publications are expected to be completed in the winter and spring of 2018.

## Discussion

### Anticipated Results

Based on previous pilot data collected by our team [11,15,23,25], we anticipate excellent levels of feasibility, acceptability, and satisfaction among participants in the in-person groups. In a study by Chadi and colleagues [15] conducted in 19 adolescent females with chronic pain, using a similar MBI, the rate of attrition was low (17%), attendance was high (84%), and participants reported positive changes in the way they coped with their condition. We anticipate similar results in the eHealth groups, based on data from adult studies [18]. We hypothesize that both the in-person and eHealth modes of delivery will provide similar and significant improvements in mindfulness skills, mood, anxiety, self-esteem, and illness perception when compared to wait-list controls and that the magnitude of these effects will be correlated with the regularity of home practice. We project that participants in in-person and eHealth groups will have similar levels of home practice. In addition, we anticipate a significant reduction in pre-post session (5:30 PM to 7:00 PM) stress levels for both in-person and eHealth groups. Finally, we anticipate that all participants will see benefits of having incorporated mindfulness in their everyday life, such as improved level of functioning, sleep, or coping with illness, which will be captured by our semistructured interviews.

### Strengths and Limitations

Anticipated difficulties described in previous studies of MBIs in clinical youth populations [26,51] such as recruitment and retention will be addressed in several ways: an efficient referral process, individual intake meetings with participants, regular communication with participants via phone and email, and incentive measures (gift cards) at project completion.

An important limitation of this study will be the small size of the sample, which was determined by several factors including funding constraints and recruitment capacity at the study site. Nevertheless, the information provided by the wide range of outcome measures and the 2 complementary approaches of the mixed methodology will provide clinically relevant information related to the eHealth delivery of MBIs in adolescents with chronic health conditions. Another limitation will be that mindfulness is challenging to measure quantitatively as a single construct. The authors are aware of some level of critique [52] that has been formulated about the MAAS scale but believe that this scale remains the best measure to test the hypothesis that the eHealth and in-person modes of delivery of the MBI are comparable in terms of teaching mindfulness content and skills.

This study will help evaluate the effectiveness of an MBI delivered to teens via technology—a highly relevant medium to connect with adolescents given their overall affinity for electronics and the Internet. Definitive results from this study will help elucidate effective ways to reach adolescents with chronic illnesses who have mobility issues or are geographically remote from urban centers, an important step in research aiming to explore how teens can integrate mindfulness in their everyday life on a long-term scale.

## Appendix

Multimedia Appendix 1 Scientific peer review and acceptance letter from Mind and Life Institute.

## Abbreviations

DASS-21: Depression Anxiety Stress Scale
MAAS-A: Mindful Attention Awareness Scale for Adolescents
MARS-A: Mindful Awareness and Resilience Skills for Adolescents
MBI: mindfulness-based intervention
MBCT: mindfulness-based cognitive therapy
MBSR: mindfulness-based stress reduction
MBSR-T: mindfulness-based stress reduction for teens
