# Supplementary material for: In-Person Versus eHealth Mindfulness-Based Intervention for Adolescents With Chronic Illness: Protocol for a Randomized Controlled Trial
Source: JMIR Res Protoc. 2017 Nov 27;6(11):e241. doi: 10.2196/resprot.7700 (PMC5725624; doi:10.2196/resprot.7700)
Supplement: Multimedia Appendix 1 [file resprot_v6i11e241_app1.pdf]

MIND & LIFE
INSTITUTE

His Holiness the XIV Dalai Lama
*Honorary Chairman*

Susan Bauer-Wu, PhD
*President*

**Board of Directors**

Richard J. Davidson, PhD
*University of Wisconsin*

Raymond J. Gellein, Jr., MBA
*Strategic Hotels & Resorts, Inc.*
*Mind and Life Vice Chair*
*Mind and Life Treasurer*

Daniel Goleman, PhD
*Psychologist, Author*

Joan Halifax, PhD
*Upaya Zen Center*

Barry Hershey, MFA
*Filmmaker*

Carolyn Jacobs, PhD
*Smith College School for Social Work, Dean Emerita*

Thupten Jinpa, PhD
*Institute of Tibetan Classics*
*Mind and Life Chair*

Bennett M. Shapiro, MD
*Merck Research Laboratories (Retired)*

Tania Singer, PhD
*Max Planck Institute for Human Cognitive and Brain Sciences*

Aaron Stern
*Academy for the Love of Learning*

Diana Chapman Walsh, PhD
*Wellesley College, President Emerita*

Via Email: nicholas.chadi@sickkids.ca

May 5, 2016

Dear Nicholas:

Thank you for submitting your application for the Mind & Life Institute's 2016 cycle of the 1440 Awards. The Review & Selection Committee has finished carefully reading and evaluating the competitive pool of proposals that were submitted this year. **I am delighted to inform you that your research application has been accepted for funding!** While the proposal has been accepted in its current form, we have attached reviewer feedback, which may help to further extend and improve your work. Overall, we were impressed by the intellectual rigor and creativity represented in your proposal and we hope your study is rewarding and productive. We look forward to learning of the results.

This award is to be used for the purposes and time period outlined in your application and the grant will be for **$15,000.** You will be contacted by the 1440 Awards Grants Manager, Sunanda Markus, shortly with further information.

On behalf of the Mind and Life Board of Directors and the 2016 Mind and Life Review & Selection Committee, please accept our heartfelt congratulations on your 1440 Award.

With best wishes,

Wendy Hasenkamp

Wendy Hasenkamp, PhD
Senior Scientific Officer
Mind & Life Institute
wendy@mindandlife.org
